# A Compact Linear Microstrip Patch Beamformer Antenna Array for Millimeter-Wave Future Communication

**DOI:** 10.3390/s24134068

**Published:** 2024-06-22

**Authors:** Muhammad Asfar Saeed, Emenike Raymond Obi, Augustine O. Nwajana

**Affiliations:** 1 School of Engineering, University of Greenwich, Chatham Maritime, Kent ME4 4TB, UK; 2 Raysoft AssetAnalytics, Regina, SK S4N 7S1, Canada; ray.obi@raysoft-aa.net

**Keywords:** 5/6G, millimeter, CST, RO3003, beamforming, substrate, EM waves, Ka band

## Abstract

5/6G is anticipated to address challenges such as low data speed and high latency in current cellular networks, particularly as the number of users overwhelms 4G and LTE capabilities. This paper proposes a microstrip patch antenna array comprising six radiating patches and utilizing a microstrip line feeding technique to facilitate the compact design crucial for 5G implementation. ROGER 3003, chosen for its advanced and environmentally friendly features, serves as the dielectric material, ensuring suitability for 5G and B5G applications. The designed antenna, evaluated at a resonating frequency of 28.8 GHz with a −10 dB impedance bandwidth of 1 GHz, offers a high gain of 9.19 dBi. Its compact array, cost-effectiveness, and broad impedance and radiation coverage position it as a viable candidate for 5G and future communication applications.

## 1. Introduction

Over the past twenty years, wireless communication has experienced remarkable advancements due to the continuous evolution of modern communication systems, largely driven by the expansion of antenna design. The research community asserts that microstrip patch antennas and millimeter waves (mm waves) stand as suitable choices to meet the demands of emerging technologies such as 5G, IoT, air traffic control, earth detection, space exploration, remote sensing mapping, autonomous vehicles, and biomedical applications in the future.

The imminent arrival of 6G necessitates the incorporation of five pivotal technologies to empower the upcoming sixth-generation communication networks: small cells, full-duplex, beamforming, MIMO, and mm waves. In the sphere of wireless communications, antenna arrays have gathered heightened attention, establishing array antenna technology as a fundamental aspect of electrical engineering. Given the swift technological advancements in modern radio systems, particularly for the impending deployment of 6G networks operating at sub-centimeter and millimeter-wave frequencies, the adoption of antenna arrays become imperative to meet progressively stringent requirements in terms of architectural complexity, broadband behavior, high-gain multi-beam characteristics, and minimal scan losses [1,2].

In the literature, various techniques have been employed to achieve beamforming [3,4,5,6]. One commonly used approach involves extending the element pattern of the array to enhance coverage [3]. Another method utilizes a 16 × 16 array technique with metal walls [4], while pattern-reconfigurable techniques have been effective in expanding scanning coverage for phased arrays [5,6]. Shared coupling among radiating elements in arrays has also been utilized to achieve large-angle beamforming in linear and phased arrays [7,8].

Additionally, previous methods have included adding a dielectric sheet above the array [9], using multilayers as a ground plane [10], and combining or mixing different techniques, including mechanical and electrical combinations, to improve beamforming [11]. While tightly coupled techniques have been successful in wideband antenna applications [12,13], they may not be as suitable for future 6G and B6G (beyond 6G) due to strong coupling between array elements. However, these methods may face limitations such as complex structures, beam scanning accuracy, efficiency, and fluctuations in gain, which need to be addressed. The advent of millimeter-wave technology has ushered in a new era in wireless communication, promising unprecedented data rates and enhanced connectivity for future networks. In this context, the design and implementation of compact and efficient antennas become paramount. This research paper introduces a novel approach for a compact linear microstrip patch beamformer antenna array tailored for millimeter-wave frequencies. This antenna array aims to address the challenges and requirements of future communication systems, offering a combination of compactness, linearity, and beamforming capabilities essential for realizing the potential of millimeter-wave communication technologies.

The main contributions of the work presented in this paper are as follows:

- Firstly, a considerably simplified design while prioritizing cost-effectiveness to reduce manufacturing intricacies. This entailed refraining from using inset cuts, slots, electroplated vias, extra parasitic patches, or any flawed ground structures that might hinder or restrict RF performance at higher frequencies. The microstrip edge-feed technique is also utilized, ensuring easy antenna measurements and smooth integration with RF circuitry.
- Secondly, the introduction of an approach involving rectangular patch elements within a series-fed topology. This innovative linear array method has notably improved the impedance bandwidth, achieving a substantial fractional bandwidth (FBW) that spans the entire 21.87% FBW of the 28.8 GHz Ka band. Simultaneously, the design preserves high flat gain characteristics.
- Thirdly, the realization of a design with an overall compact size of 7.5 × 22 mm and simple linear structured radiating elements responsible for observing constructive and destructive interference.

## 2. Antenna Design and Development

The proposed antenna design structure consists of a rectangular sheet of dielectric material measuring 26 mm × 6 mm (with a dielectric constant of 3, a loss tangent of 0.0009, and a thickness of 1.574 mm). This sheet is coated by a conducting ground plane and contains six radiating patch elements on top of it.

The antenna employs microstrip line feed to connect the six radiating patches for proper transmission of current between the radiating patches. The input impedance of each patch is influenced by the series-fed microstrip transmission line (MTL) and the adjacent patch. Initially, the impedance of a single patch element was measured to be approximately 210 Ω. By connecting the initial patch element to the other five optimized patches and their respective MTLs, the impedance characteristics of each patch element change along with varying phases. This design approach results in the formation of beams angled differently in various directions.

Consequently, the antenna array achieves a broad −10 dB impedance matching while maintaining an average input impedance of 50 Ω overall. The series MTL is optimized with a width of 0.27 mm (within fabrication limits) and a length (Ls) of 3 mm. A microstrip line feed technique is utilized to feed the radiating patches. This technique was employed for ease of fabrication and enhanced bandwidth. In comparison with other feeding techniques, microstrip line feed offers the required features like higher bandwidth and ease of fabrication, in addition to the benefit that it can be fabricated on the same substrate [14]. A full ground plane was used at the bottom of the substrate to achieve a broadside radiation pattern.

The choice of Roger RO3003 as the substrate material is motivated by its advantageous characteristics, including accessibility, low cost, stability in diverse environments, and superior high-frequency properties with lower losses at higher frequencies [15,16]. The relatively high permittivity of Roger 3003 allows for compact antenna designs, enabling a reduction in the physical size of the microstrip patch elements in the array. Roger 3003 substrate typically has a low dielectric loss, which minimizes signal attenuation and ensures efficient transmission, particularly at higher frequencies. The combination of low dielectric loss and high permittivity contributes to a broader bandwidth, allowing the antenna array to operate over a wider range of frequencies.

**Figure 1:**
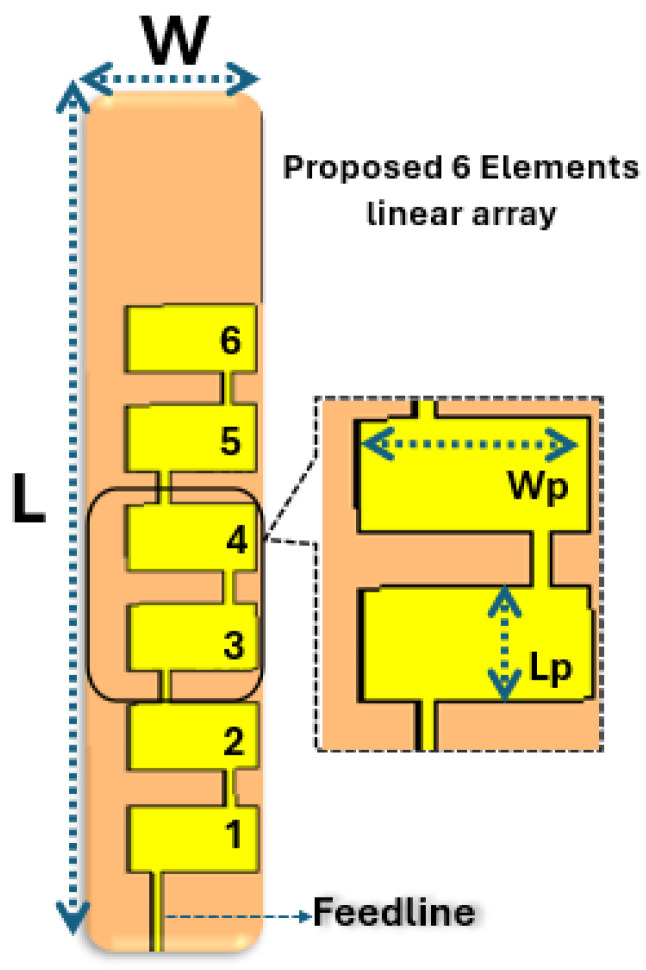
Structure of the linear MPA array.

Roger 3003 substrate exhibits good mechanical stability, which is important for maintaining the structural integrity of the antenna array, especially in applications where the antenna may be subject to mechanical stress. Roger 3003 is often chosen for its balance of performance and cost, making it a cost-effective choice for various applications without compromising on essential electrical characteristics. The substrate’s properties facilitate ease of fabrication, making it suitable for mass production and deployment in commercial antenna systems. Figure 1 depicts the composition of the designed antenna, while Table 1 outlines its physical measurements. The rectangular radiating elements placed above the substrate are connected through a thin microstrip transmission line. Each segment of the feedline linking the radiating elements and the primary source has dimensions of 2600 µm in length and 300 µm in width. The ground plane is positioned beneath the substrate, measuring 26 mm × 6 mm. The balanced feed points for each antenna are located horizontally, as this configuration yielded optimal return loss results. Microstrip patch antennas inherently exhibit narrow bandwidth [17]. One approach to overcoming this limitation is to increase substrate thickness, simultaneously reducing the quality factor to enhance impedance bandwidth. However, this solution introduces drawbacks such as heightened surface waves, larger antenna dimensions, and increased losses. These trade-offs, including alterations to the radiation pattern and potential interference with other electronics on the substrate, make it unfavorable, particularly in the 28.8 GHz band. Therefore, instead of confining a single patch antenna element to a narrow frequency range, our strategy involves increasing the number of impedance resonances within the desired band, achieving broad impedance matching in the 28–30 GHz range. The collective effect of multiple patch elements in the array results in higher overall gain compared to a single antenna element. Beamforming with a uniform linear array helps minimize interference and increases the signal-to-noise ratio (SNR), leading to improved overall signal quality [18,19,20,21,22,23]. A uniform linear microstrip patch antenna array, shown in Figure 1, is advantageous for beamforming applications due to its ability to enhance gain, improve signal quality, and adapt to changing conditions.

## 3. Analysis of the Designed Array

The performance of the linear microstrip patch antenna is evaluated through various free-space parameters, including the matching of the transmission line, S-parameters, or return loss, which provide insights into the resonant frequency and current distribution, as well as the far-field patterns in both the E-plane and H-plane and the overall gain. A directional beam of a microstrip patch antenna array with six radiating patches was achieved through careful designing and configuration of the antenna elements. The layout of the radiating patches was designed to create constructive interference in a specific direction while minimizing radiation in other directions. This is typically achieved by selecting the rectangular dimensions and positions of the patches relative to each other. The radiating patches are connected to each other using a thin feedline. The dimensions of the designed antenna array are mentioned in Table 1.

The design of a microstrip patch antenna array is explored by systematically increasing the number of radiating patches from one to six to achieve the desired performance goals. The initial design involves a single radiating patch antenna. This serves as the baseline for comparison and helps in understanding the fundamental characteristics of the rectangular microstrip patch antenna, such as impedance matching, radiation pattern, reflection coefficient, and gain. The next step involves expanding the antenna array to three rectangular radiating patches. Mutual coupling in a three-element antenna array causes interference and performance degradation due to electromagnetic interactions between elements. It is reduced by increasing element spacing. Increasing the distance between adjacent antenna elements reduced the strength of the mutual coupling. Typically, a spacing of at least 0.5 to 1 wavelength is recommended to minimize interaction; the spacing between elements is half-wavelength. Moreover, this allows for investigating the impact of adding more elements on the antenna’s performance by constructive and destructive interference of the propagated EM wave. By carefully designing the spacing and configuration of the patches, improvements in gain, directivity, and bandwidth were achieved.

**Figure 2:**
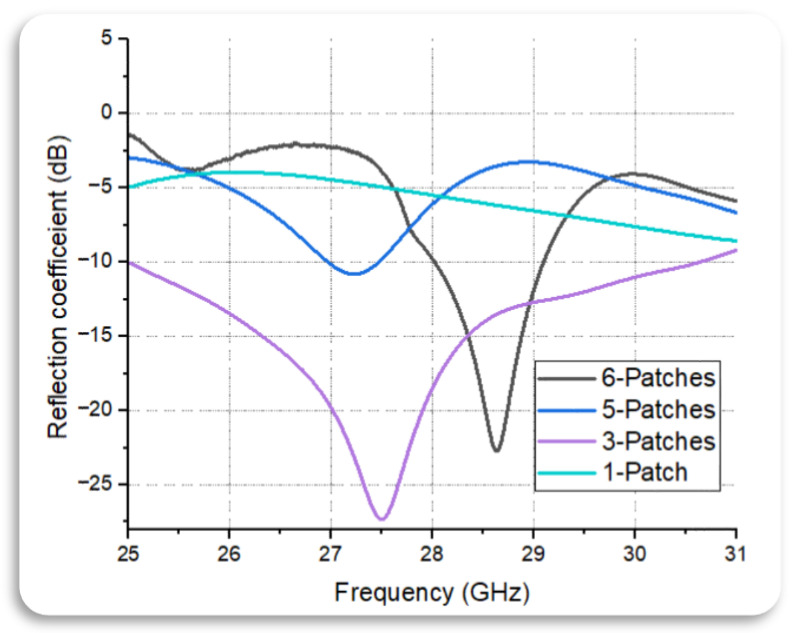
Reflection coefficient from integrating different numbers of series-fed patch antenna elements (1, 3, 5, and 6 elements).

The antenna array was further expanded to five radiating patches. This step explored the trade-offs between complexity and performance, as adding more patches led to increased directivity and gain while also introducing challenges, such as issues with mutual coupling and impedance matching. These issues were overcome by appropriate design optimization using six radiating patches to meet the desired performance requirements. Through design iterations and optimization techniques and placing rectangular patches 0.15 mm apart in a linear pattern, the antenna array is fine-tuned to achieve the desired radiation characteristics, such as directional beamforming, high gain, wide bandwidth, and efficient impedance matching. Figure 2 shows the reflection coefficient from integrating different numbers of series-led patch antenna elements.

### 3.1. Simulated Results

#### 3.1.1. Reflection Coefficient

**Figure 3:**
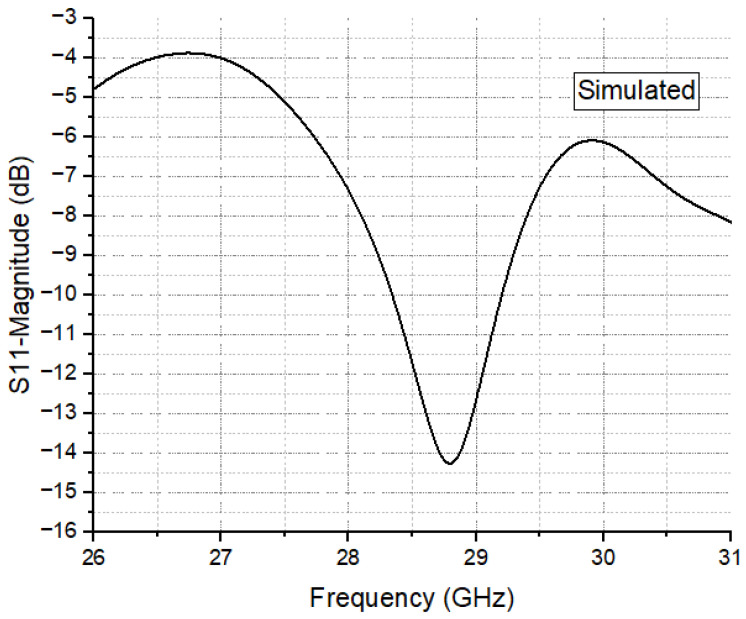
Simulated reflection coefficient of the proposed antenna.

The optimized simulated reflection coefficient (with −10 dB impedance bandwidth) and achieved gain of the proposed 1 × 6 array are depicted in Figure 3. The −10 dB impedance bandwidth spans from 28 GHz to 29.5 GHz, covering the 28 GHz band. At 28.5 GHz, the array attains a peak gain of 9.19 dBi within the desired band of interest. For an input signal of 0.5 W, the accepted power remains above 0.42 W throughout the 28–30 GHz range.

The radiated power fluctuates between 0.38 W and 0.42 W, resulting in a radiation efficiency exceeding 84.81% and a total efficiency exceeding 81.26% across the entire 28 GHz band. It is important to note that radiation efficiency represents the ratio of radiated power to accepted power, while total efficiency factors in losses due to mismatch by considering the ratio of radiated power to input stimulated power.

In Figure 3, the operating band is depicted, ranging from 28.2 GHz to 29.2 GHz, with the antenna shown to radiate at 28.8 GHz. Additionally, the figure highlights that the proposed antenna not only widens the bandwidth but also effectively supports communication operations in the Ka band of the millimeter-wave frequency range for 5G and B5G.

The simulated results presented in Figure 3 confirm the resonance of the antenna at 28.8 GHz, with a −10 dB impedance bandwidth of 1 GHz and a return loss below −10 dB, validating its resonance within the Ka band.

#### 3.1.2. Current Distribution

**Figure 4:**
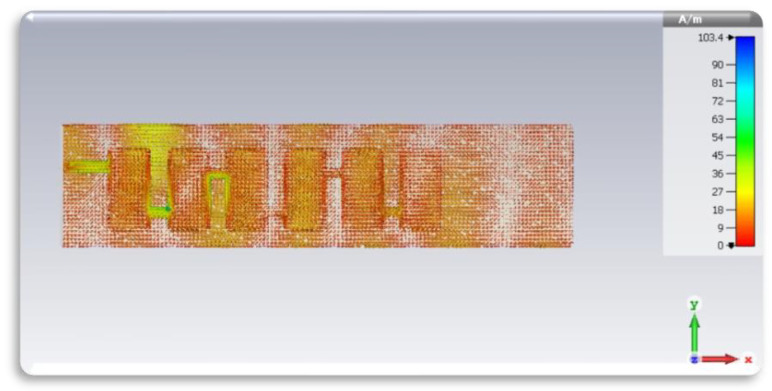
The current distribution of the designed antenna array.

Figure 4 depicts the current distribution of the antenna, showing a traveling wave pattern primarily generated by the middle section of the antenna design, with radiation originating from the microstrip line feeding port.

Additionally, Figure 4 illustrates the current distribution along the radiating patches of the antenna design. At the resonant frequency of the antenna, a significant portion of the current flows along the patch’s edges. This edge current contributes to radiation, producing the desired electromagnetic field pattern. This also illustrates a portion of the current flows, which is radially outward from the feed point along the patch’s surface. This current distribution helps excite the desired resonance mode and contributes to the radiation pattern.

Moreover, with standing waves on the patch surface of the microstrip patch antenna array, the current distribution varies along the length and width of the patch. This distribution is influenced by factors such as the patch dimensions, substrate properties, and excitation method. The antenna is properly matched at 50 Ω impedance.

#### 3.1.3. Impedance Matching

Impedance matching is crucial for optimizing power transfer from the source to the radiating patches of the antenna. When the impedance of the microstrip line precisely matches that of the radiating patches, signal reflection is minimized, ensuring maximum power delivery to the patches via the transmission line. This optimization enhances signal efficiency and minimizes losses along the microstrip line. Conversely, mismatches in impedance lead to signal reflections, resulting in loss and degradation of system performance. Achieving a 50 Ω impedance match minimizes signal loss, improving signal integrity and enabling higher gain, measured at 9.61 dBi in this case.

Furthermore, impedance matching facilitates a broader bandwidth of operation. Well-matched microstrip lines can support a wider range of frequencies, as demonstrated by the antenna’s achieved bandwidth of 0.55 GHz within the sub-terahertz frequency range.

The microstrip line feeding technique is utilized to feed the antenna, which is perfectly matched. The simulated results verify the matching of the antenna. The dimensions of the transmission line are explicit in Table 1.

**Figure 5:**
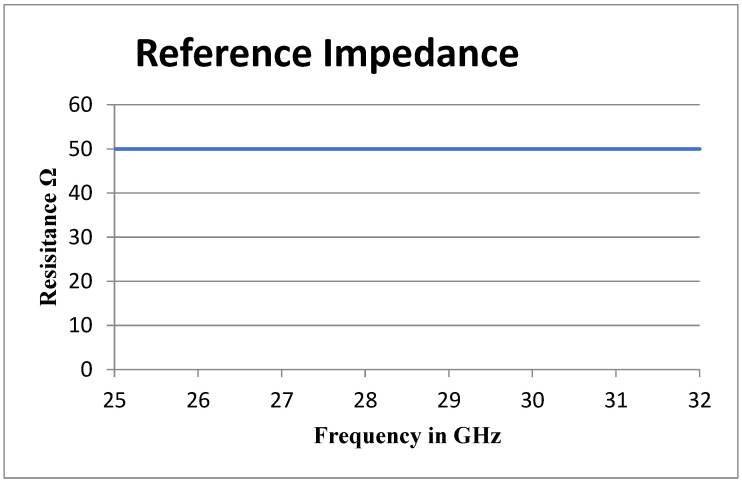
Reference impedance.

Impedance matching helps maintain a uniform radiation pattern across all the six radiating elements of the antenna array. Figure 5 shows that the reference impedance for the proposed antenna array is matched at 50 Ohms. Mismatched impedance can cause variations in the radiation pattern, leading to distortions or irregularities in the desired coverage area. As the designed microstrip line of the antenna is in proper impedance matching, this reduces the likelihood of electromagnetic interference (EMI) from nearby sources or other antennas. By optimizing the calculated values of the impedance, the antenna array turned better to reject unwanted signals and improved its signal-to-noise ratio (SNR).

#### 3.1.4. Radiation Pattern

**Figure 6:**
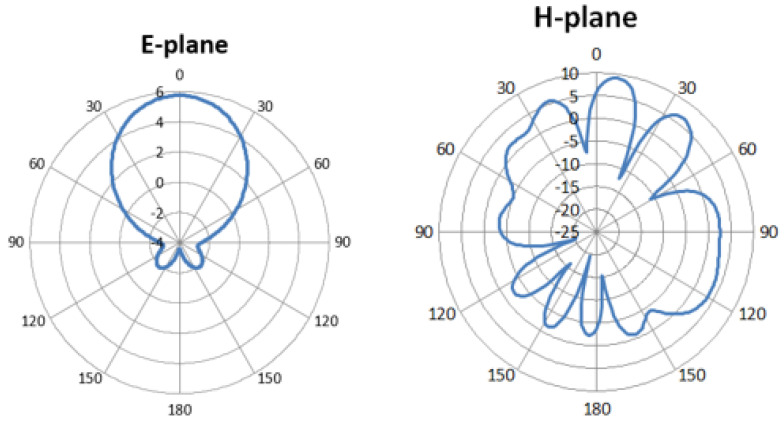
Two-dimensional far-field patterns of the designed antenna.

**Figure 7:**
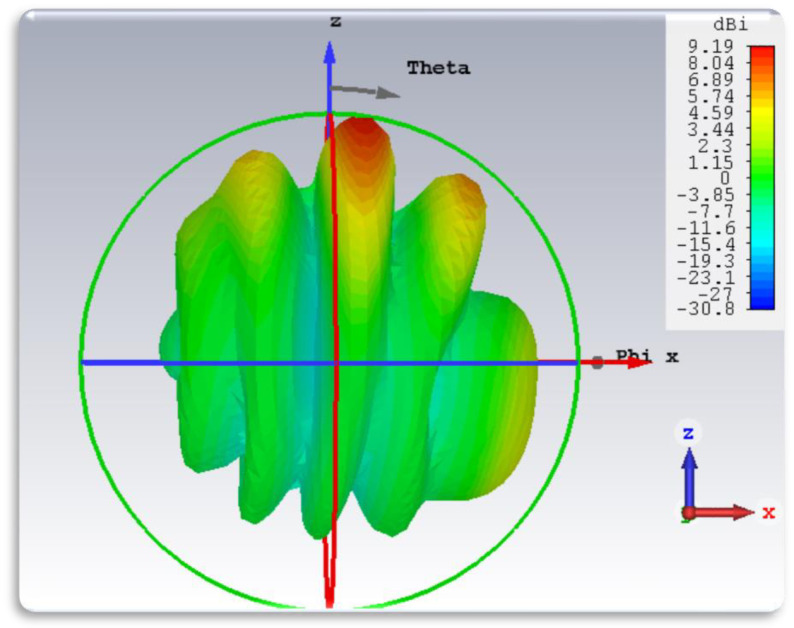
Three-dimensional radiation pattern of the designed antenna.

Figure 6 illustrates the 2D far-field radiation pattern of the antenna in the ϕ = 0° and ϕ = 90° planes, respectively, while Figure 7 displays the corresponding 3D radiation pattern. The simulated results of both 2D and 3D radiation patterns demonstrate the antenna’s favorable radiation coverage in both planes. The main lobe exhibits a magnitude of 5.75 dBi, with an angular width (3 dB) measured at 84.6°. The main lobe forms a beam shape, as depicted in the polar plot. The 3D radiation patterns at various frequency points are depicted in Figure 7.

A fan-shaped radiation pattern is observed, with a wider half-power beamwidth (HPBW) in the *x*-*z*-plane (φ = 0°) ranging from 63.44° to 77° across 28 GHz. Conversely, a relatively narrow HPBW is attained in the *y*-*z*-plane (φ = 90°), ranging from 12° to 14°, as shown in Figure 6.

This phenomenon arises from the linear geometry of the array, where the radiation pattern is compressed along the direction of the elements while expanding in the orthogonal direction. Fan beam patterns provide wide coverage in one dimension and are more efficient in concentrating power along a narrow beam in one dimension. This type of beam pattern allows for efficient use of transmitted power, maximizing the signal strength in the desired direction while minimizing energy wastage in other directions.

#### 3.1.5. Gain and Efficiency

**Figure 8:**
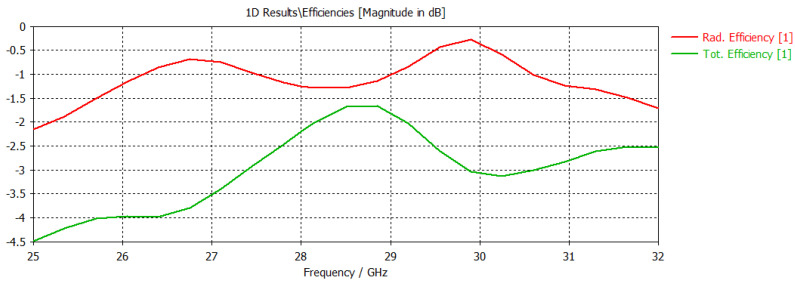
Efficiency of the designed antenna array.

At the resonant frequency, the designed antenna exhibits an impressive gain profile, reaching a value of 9.19 dBi. Additionally, the antenna’s efficiency surpasses the acceptable level. The radiation efficiency of the antenna is measured to be 65% at 28 GHz. This high efficiency suggests minimal power losses, ensuring that most of the input power is effectively radiated. The efficiency curve presented in Figure 8 shows stable performance across the operational bandwidth, indicating reliable and energy-efficient operation. The gain of the designed antenna is illustrated in the 3D far-field plot of the graph shown in Figure 7. The performance comparison of the proposed antenna array to related existing literature is presented in Table 2.

## 4. Prototype Fabrication and Analyzed Results

**Figure 9:**
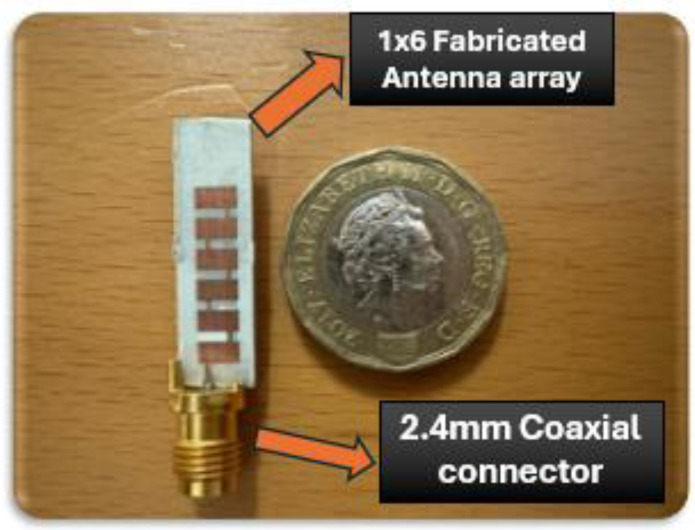
Fabricated prototypes of the proposed 28 GHz linear MPA arrays.

The proposed antenna array was fabricated on the substrate sheet layered with copper at the top and bottom; the copper cladding is 0.5 mm, and the substrate thickness is 1.574 mm. The fabricated prototypes of the proposed 1 × 6 array are presented in Figure 9.

**Figure 10:**
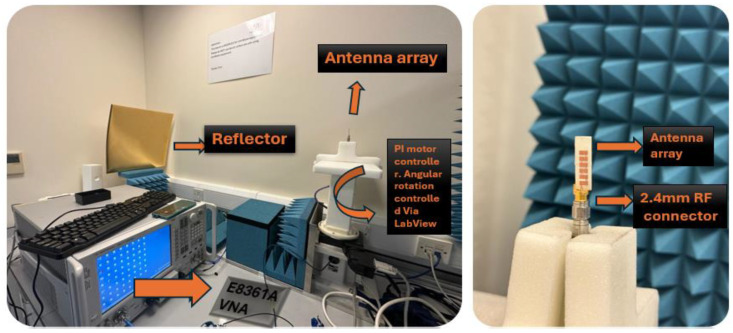
Measurement setup for measured results of linear MPA array.

Excitation of the antenna array was achieved using an edge-launched reusable 2.4 mm standard V-connector. The measurement setup, shown in Figure 10, employs 2.4 mm standard RF equipment with a maximum frequency support of 40 GHz. Reflection coefficients (i.e., −10 dB impedance BW) of the proposed 1 × 6 array were measured utilizing an Agilent E8361A vector network analyzer (VNA).

**Figure 11:**
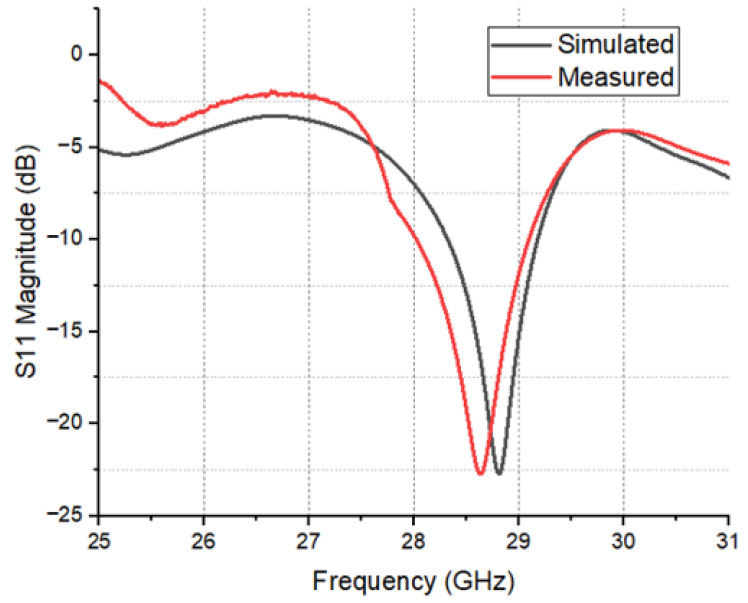
Reflection coefficient of the proposed antenna array.

**Figure 12:**
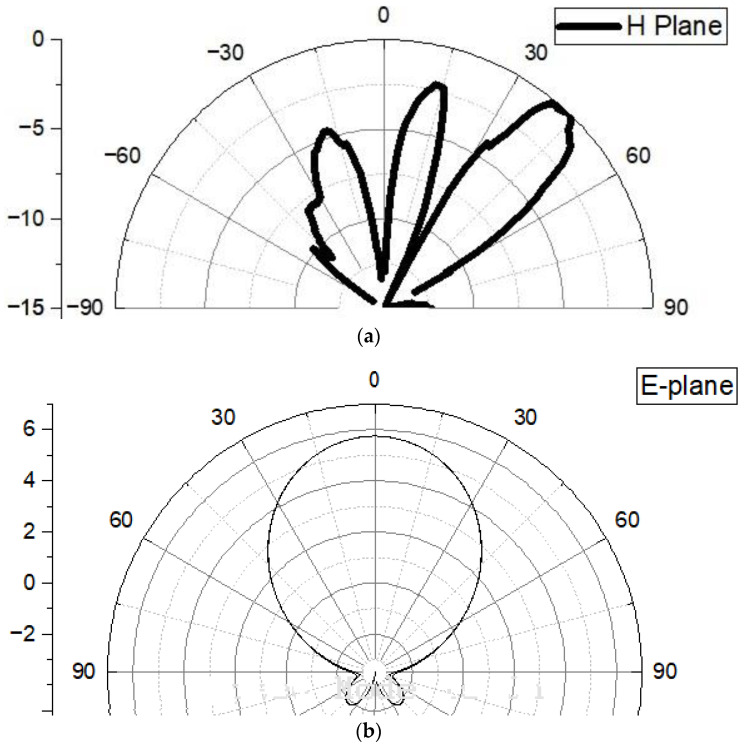
Measured far-field radiation patterns of the designed antenna array: (**a**) H-plane; (**b**) E-plane.

The designed linear MPA array measured reflection coefficient results are illustrated in Figure 11. It is evident from Figure 11 that the designed antenna array covers an acceptable bandwidth of 700 MHz at the resonant frequency. The far-field radiation pattern of the designed microstrip patch antenna at 28 GHz is presented. The polar plot in Figure 12 illustrates the radiation intensity as a function of angle, where the main lobe is directed at 55 degrees with a beamwidth of 35 degrees at −3 dB points. The peak gain of 9.19 dBi is achieved, as indicated by the highest point on the plot. Side lobes are observed relative to the main lobe, demonstrating good directivity and minimal undesired radiation. These results are consistent with the design objectives and suggest the antenna’s suitability for future communication application

The measured results exhibit close correspondence with simulations, with minor discrepancies attributed to fabrication tolerance and surface roughness. Furthermore, an over-the-air (OTA) antenna measurement setup was established in an open space within an indoor lab environment to capture far-field radiation patterns and gain, as depicted in Figure 10.

## 5. Conclusions

In conclusion, this research successfully demonstrates the design and performance evaluation of a microstrip patch antenna array optimized for 5G and future communication applications. Through systematic experimentation and analysis, the antenna array comprising six radiating patches and utilizing microstrip line feeding techniques achieves significant milestones, including a resonant frequency of 28.8 GHz with a −10 dB impedance bandwidth of 700 MHz and a high gain of 9.19 dBi. The use of ROGER 3003 as the dielectric material proves effective, ensuring broad impedance and radiation coverage essential for modern communication needs. The compact design, cost-effectiveness, and environmental suitability of the antenna make it a compelling choice for integration into 5G networks and beyond. This study underscores the potential of microstrip patch antenna arrays in advancing wireless communication technologies.

## Figures and Tables

**Table 1:** Dimensions of the designed linear MPA array.

Antenna Elements	Parameters	Dimensions (mm)
Substrate	Length, L	26
	Width, W	6
	Thickness, t	1.574
	Permittivity, Ɛ	3
Feedline	Feedline length, FL	2.6
	Feedline width, WL	0.3
Ground	Ground length, GL	26
	Ground width, WL	6
Patch	Patch length, PL	2
	Patch width, WL	4

**Table 2:** Comparison of designed MPA array.

Ref.	Size (mm)	Resonant Frequency (GHz)	Bandwidth (MHz)	Gain (dB)
[9]	42.5 × 42.5	28	600	5
[17]	102 × 86	28	1500	1.51
[10]	2.5 × 0.53	52	1350	3.5
[18]	37 × 37	28	2400	9
[23]	7 × 29.50	18	700	7.51
[19]	13 × 23	64	1350	19.23
Proposed work	6 × 26	28.8	700	9.19

## Data Availability

Data are contained within the article.
